# Dynamic LVEF Decline and Serum NT-proBNP and Uric Acid Levels before Heart Transplantation are Independent Predictors of Adverse Outcomes in Young Adult Patients with Dilated Cardiomyopathy

**DOI:** 10.31083/j.rcm2505153

**Published:** 2024-04-30

**Authors:** Jian Li, Shouling Mi, Meng Wang, Mengwan Li, Qilong Guo, Fan Yang, Junhua Ge

**Affiliations:** ^1^Department of Cardiology, Qingdao Municipal Key Laboratory of Hypertension (Key Laboratory of Cardiovascular Medicine), The Affiliated Hospital of Qingdao University, 266000 Qingdao, Shandong, China; ^2^Department of Cardiology, Shanghai Institute of Cardiovascular Diseases, National Clinical Research Center for Interventional Medicine, Zhongshan Hospital, Fudan University, 200032 Shanghai, China; ^3^Department of Cardiology, Guizhou Provincial People’s Hospital, 550002 Guiyang, Guizhou, China

**Keywords:** dilated cardiomyopathy, heart transplantation, NT-proBNP, uric acid, ventricular remodeling, young adults

## Abstract

**Background:**

The present study investigated the predictors of adverse outcomes in young adult patients with dilated cardiomyopathy (DCM) who underwent heart transplantation (HTx).

**Methods:**

Twenty-four young adult patients (aged 18–45 years) with DCM who underwent HTx in our hospital from January 2012 to December 2022 were included in this retrospective analysis. Pre- and post-HTx data were collected for echocardiography, N-terminal pro-brain natriuretic peptide (NT-proBNP), and uric acid (UA). Data collected at the time of DCM diagnosis were designated as baseline data. Post-HTx assessments were conducted at 1 week and 3, 6, 12, and 36 months post-HTx. The primary endpoint was defined as any adverse event, including left ventricular ejection fraction (LVEF) <50% (n = 3), 50% increase in right or left ventricular diameter (n = 12), or death (n = 2). Patients were categorized into a non-adverse-event group (n = 12) or an adverse-event group (n = 12).

**Results:**

Baseline NT-proBNP (*p* = 0.014) and UA (*p* = 0.012) were significantly higher in the adverse-event group than in the non-adverse-event group. Baseline NT-proBNP >7390 pg/mL (relative risk (RR) = 7.412, *p* = 0.046), UA >542 µmol/L (RR = 8.838, 95% confidence interval (95% CI) = 1.541–50.694, *p* = 0.014), and sustained reduction in LVEF (≥3%) over a 2-year pharmacological treatment prior to HTx (RR = 3.252, *p* = 0.046) were significantly associated with an increased risk of adverse events post-HTx.

**Conclusions:**

In young adult DCM patients post-HTx, heightened baseline levels of NT-proBNP and UA levels and a sustained reduction in LVEF over time prior to undergoing an HTx are significantly associated with an increased risk of adverse events post-HTx. Future studies are needed to observe whether individualized monitoring strategies could reduce the incidence of adverse events following HTx in these patients.

## 1. Introduction

Dilated cardiomyopathy (DCM) is characterized by left ventricular (LV) chamber enlargement and systolic dysfunction in the absence of known abnormal loading conditions or significant coronary artery disease. The estimated prevalence of DCM is 1:2500 in the general population, which constitutes the third most common type of heart failure and the most frequent cause of heart transplantation (HTx) [1]. Up to 50% of patients diagnosed with DCM as children either die or undergo HTx within 5 years of the diagnosis [2].

HTx offers the best survival benefit for patients with DCM, and DCM accounts for 50% of HTx cases in Europe and the United States. Notably, DCM constitutes as much as 73.9% of HTx cases in China [3]. New York Heart Association functional class I or II could be achieved in more than 90% of patients at 1 to 3 years post-HTx [4]. Post-transplant survival has improved over time. The median survival after adult heart transplants performed between 2002 and 2009 is 12.5 years, extending to 14.8 years among 1-year survivors [5]. According to recent data from the International Society of Heart and Lung Transplantation (ISHLT) in 2014, the 1-year survival rate in heart transplant recipients is 84.5%, and the 5-year rate is 72.5% [6, 7].

Although HTx has shown satisfactory long-term outcomes, its success is hindered by challenges such as the limited availability of donor hearts and the potential for donor heart dysfunction or rejection. Notably, significant risk factors for mortality in the initial five years post-HTx encompass recipient and donor ages, pulmonary vascular resistance, donor body mass index, and the donor/recipient weight ratio [8].

Limited data exist on the use of biomarkers, such as the brain natriuretic peptide and N-terminal-pro brain natriuretic peptide (NT-proBNP), to identify adverse recipient outcomes in adults following HTx [9]. Research on echocardiographic measures and outcomes post-HTx remains underexplored. Left ventricular hypertrophy, defined by echocardiography, has been commonly observed at 1-year post-HTx and is a robust and independent predictor of increased mortality [10]. Similarly, Raichlin *et al*. [11] reported the importance of assessing LV mass by echocardiography in heart transplant recipients as a crucial prognostic indicator associated with mortality post-HTx. The research on changes in left ventricular ejection fraction (LVEF) and ventricular chamber remodeling over time post-HTx is currently limited. Furthermore, the clinical significance and related risk factors of these indicators post-HTx are poorly characterized, especially for young adult patients (aged 18–45 years) with DCM.

To address the aforementioned knowledge gap, the present study comprehensively assessed the pre- and post-HTx clinical and echocardiographic characteristics of young adult patients with DCM. Serial changes in echocardiographic measurements and important laboratory data were analyzed post-HTx over 36 months. The study aimed to identify predictors of adverse events, defined as a decrease in LVEF (<50%), enlargement of cardiac chambers (no less than 50% increase in the right ventricular diameter or left ventricular diameter), or death post-HTx in this young DCM patient cohort.

## 2. Methods

### 2.1 Study Population

This retrospective study comprised a cohort of 24 young adult patients diagnosed with DCM who underwent HTx. The study population was derived from a dataset of consecutive DCM patients (n = 67) referred to our hospital between January 2012 and December 2022. DCM was defined by the presence of LV or biventricular dilatation and systolic dysfunction, excluding coronary artery disease or valve disease sufficient to cause global systolic impairment. The age range of the participants was from 18 to 45 years. Exclusion criteria were applied to patients with ischemic or valvular etiologies of LV dysfunction, as confirmed by coronary angiography and echocardiography. Additionally, patients with identifiable contributors to systolic dysfunction, such as alcohol abuse, chemotoxicity, congenital heart disease, neuromuscular disease, or systemic conditions capable of transiently impairing systolic function, were excluded. The 24 enrolled DCM patients exhibited insufficient responses to an average 2-year pharmacological treatment, characterized by a persistent decline in LVEF. Consequently, these patients were referred to our hospital for HTx.

### 2.2 Echocardiography Measures 

As outlined previously, echocardiographic parameters were assessed in all patients using two-dimensional echocardiography at the initial hospital admission and during follow-up, adhering to the American Society of Echocardiography guidelines and the European Association of Cardiovascular Imaging [12]. In summary, LVEF was determined in the LV apical 4- and 2-chamber views using the Simpson biplane method. Measurements of end-diastolic left ventricular diameter (LVD) and end-systolic left atrial anterior–posterior diameter (LAD) were taken in the LV long-axis view. End-diastolic right ventricular middle diameter (RVD), along with end-systolic right atrial long-axis diameter (RAD1) and short-axis diameter (RAD2), were measured from a right ventricular focused apical 4-chamber view. Pulmonary systolic artery pressure (PASP) was derived from the peak tricuspid regurgitation (TR) jet velocity using the simplified Bernoulli equation in combination with an estimated right atrial pressure (RAP): PASP = ${\mathrm{4V}}^{2}$ + RAP, where V indicates the peak TR jet velocity. RAP was estimated from the inferior vena cava diameter and respiratory changes.

### 2.3 Data Collection of Baseline and Follow-up

**Fig. 1.**
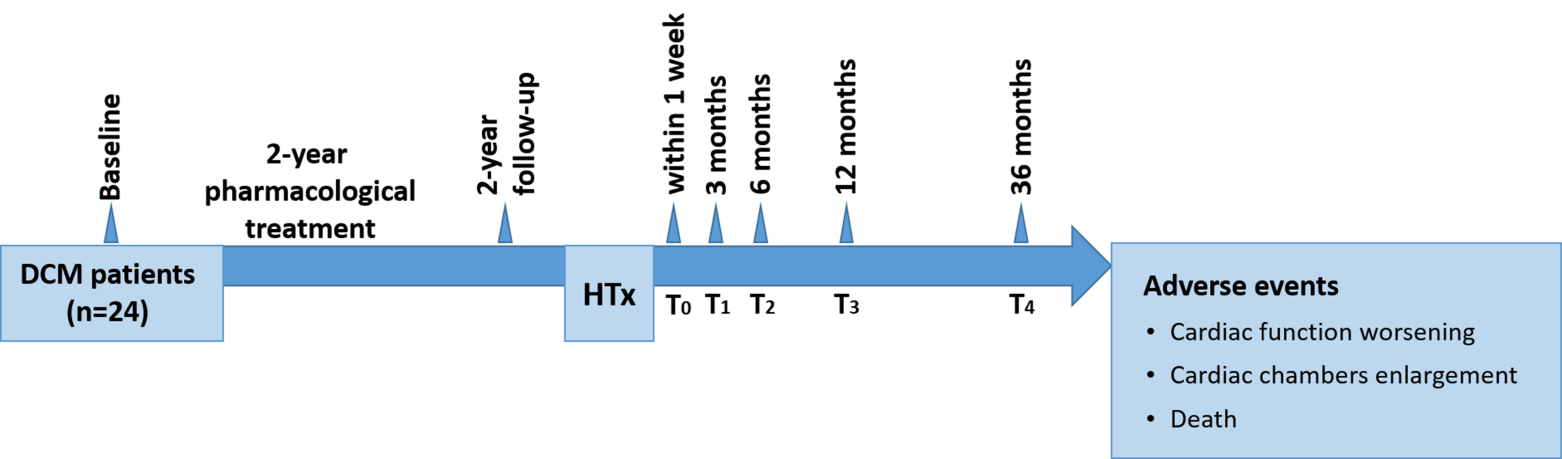
**Study flowchart**. DCM, dilated cardiomyopathy; HTx, heart transplantation.

We conducted a retrospective collection of clinical, laboratory, and echocardiographic data for HTx patients. The pre-HTx data included information collected at the time of DCM diagnosis, identified as baseline data, as well as data from the follow-up period after an average of 2-year pharmacological treatment before HTx (Fig. 1). Patients were administered standard HTx medical treatments post-HTx, according to the related guideline of the European Society of Cardiology [13]. The post-HTx assessments were scheduled at 1 week, 3 months, 6 months, 12 months, and 36 months post-HTx (Fig. 1). The collected follow-up data post-HTx included cardiac morphology and functional measures detected by echocardiography as well as the NT-proBNP and uric acid (UA) levels.

### 2.4 Study Endpoint Definition and Patient Grouping

The primary endpoint was defined as a composite of adverse events, including LVEF <50% at 36 months post-HTx, a 50% increase in RVD or LVD during the follow-up, or death. Patients were categorized into the non-adverse-event group (n = 12) and the adverse-event group (n = 12).

### 2.5 Statistical Analysis

Continuous variables are expressed as the mean ± standard deviation (SD) or median (interquartile range), and categorical variables are expressed as numbers (percent). Differences between the two groups were compared using the independent samples *t*-test or the Wilcoxon rank sum test (Mann–Whitney U test). Categorical data were compared across groups using the Chi-square test or Fisher’s exact test. One-way repeated measures analysis of variance (ANOVA) with the general linear model was conducted to compare the dynamic change in variables over time. The Youden index method was used to define optimal cutoffs of NT-proBNP and UA associated with adverse events. We employed modified Poisson Log-linear models to ascertain independent risk factors linked to adverse outcomes, reporting the relative risk (RR) and 95% confidence interval (95% CI). A *p* value < 0.05 (two-tailed test) was considered statistically significant. The statistical analysis was performed using the SPSS statistical software, version 23.0 (IBM SPSS Statistics, Chicago, IL, USA).

## 3. Results

### 3.1 Pre-HTx Clinical and Echocardiographic Characteristics and Outcome 

The mean age of the entire HTx cohort was 32 ± 7 years. Of the 24 patients, 15 (62.5%) were male. At 36 months post-HTx, 12 patients (50%) reached the primary endpoint and were included in the adverse event group. Among them, three patients had LVEF <50%, 12 experienced no less than a 50% increase in either LVD or RVD, and two patients died.

As shown in Table 1, baseline NT-proBNP (11279 (8378–17882) *vs.* 3907 (2889–8912) pg/mL, *p* = 0.014) and UA (775 (611–828) *vs.* 429 (360–762) µmol/L, *p* = 0.012) were significantly higher in the adverse-event group than those in the non-adverse-event group. Following an average of 2-year pharmacological treatments, HTx patients demonstrated an LVEF of 23.0 ± 4.5% before undergoing HTx, while the LVEF was similar between groups (22.8 ± 5.5% *vs.* 23.3 ± 2.5%, *p* = 0.827). Patients with adverse events demonstrated a significant LVEF reduction during the 2-year pharmacological treatment before HTx, while those in the no adverse-event group showed marginal or unchanged LVEF over time (percentage change: –12.0% (–22.1% to –5.7%) *vs.* 2.4% (–8.5% to 11.1%), *p* = 0.028).

**Table 1.** **Baseline and pre-HTx clinical and echocardiographic characteristics of DCM patients with and without adverse events**.

			Total	Non-adverse-event group	Adverse-event group	*p* value
			(n = 24)	(n = 12)	(n = 12)	
Baseline data (collected at the time of DCM diagnosis)						
Age (years)			32 ± 7	33 ± 6	31 ± 8	0.680
Male (n (%))			15 (62.5)	7 (58.3)	8 (66.7)	1.000
Body mass index (kg/m²)			23.3 ± 5.2	22.8 ± 4.0	23.9 ± 6.4	0.610
Hypertension (n (%))			10 (41.7)	4 (33.3)	6 (50.0)	0.408
Diabetes (n (%))			0 (0.0)	0 (0.0)	0 (0.0)	–
Hypercholesterolemia (n (%))			1 (4.2)	1 (8.3)	0 (0.0)	1.000
Chronic kidney disease (n (%))			0 (0.0)	0 (0.0)	0 (0.0)	–
Smoking (n (%))			9 (37.5)	4 (33.3)	5 (41.7)	1.000
Drinking (n (%))			3 (12.5)	1 (8.3)	2 (16.7)	1.000
Medications (n (%))						
	Furosemide		23 (95.8)	11 (91.7)	12 (100)	1.000
	Spironolactone		23 (95.8)	11 (91.7)	12 (100)	1.000
	Sacubiril/valsartan		3 (12.5)	1 (8.3)	2 (16.7)	1.000
	Beta blocker		24 (100)	12 (100)	12 (100)	–
	ACEI		7 (29.2)	5 (41.7)	2 (16.7)	0.371
	Digoxin		20 (83.3)	12 (100)	8 (66.7)	0.093
Laboratory data						
	NT-proBNP (pg/mL)		8645 (3846–14,186)	3907 (2889–8912)	11,279 (8378–17,882)	0.014
		>7390 pg/mL	14 (58.3)	3 (25.0)	11 (91.7)	0.003
	cTNI (ng/mL)		0.02 (0.01–0.03)	0.02 (0.01–0.03)	0.01 (0.01–0.03)	0.410
	CRP (mg/L)		4.90 (3.24–11.96)	9.52 (3.45–18.24)	4.35 (2.53–5.62)	0.052
	AST (U/L)		22.0 (17.3–28.8)	20.0 (14.5–23.0)	24.0 (21.3–31.3)	0.060
	ALT (U/L)		22.5 (19.0–28.5)	21.0 (17.5–23.8)	22.5 (19.5–39.8)	0.219
	Cr (µmol/L)		70.0 (54.3–89.5)	80.5 (61.3–91.5)	64.0 (52.3–72.8)	0.128
	TG (mmol/L)		1.22 (0.81–1.65)	1.39 (0.75–2.65)	1.11 (0.83–1.53)	0.378
	TC (mmol/L)		4.12 (3.32–6.61)	3.81 (3.22–6.03)	4.75 (3.69–7.04)	0.143
	LDL-C (mmol/L)		2.91 (2.07–3.89)	2.80 (2.07–3.84)	3.09 (1.92–4.32)	0.671
	UA (µmol/L)		658 (422–820)	429 (360–762)	775 (611–828)	0.012
		>542 µmol/L	15 (62.5)	4 (33.3)	11 (91.7)	0.009
Echocardiography						
	LVEF (%)		24.9 ± 5.3	23.1 ± 5.7	26.7 ± 4.3	0.096
	LVD (mm)		68.1 ± 4.9	67.1 ± 5.7	69.1 ± 3.9	0.328
	RVD (mm)		45.6 ± 13.4	46.8 ± 15.3	44.4 ± 11.7	0.668
	LAD (mm)		50.5 ± 8.6	50.7 ± 7.9	50.3 ± 9.5	0.926
	RAD1 (mm)		59.0 ± 8.7	58.0 ± 10.0	60.1 ± 7.5	0.570
	RAD2 (mm)		46.4 ± 6.2	45.2 ± 3.5	47.7 ± 8.1	0.342
	PASP (mmHg)		46.7 ± 11.4	45.0 ± 6.4	48.4 ± 14.9	0.477
Pre-HTx data (collected over a 2-year pharmacological treatment before HTx)						
Echocardiography						
	LVEF (%)		23.0 ± 4.5	22.8 ± 5.5	23.3 ± 3.5	0.827
	LVD (mm)		73.5 ± 3.9	72.9 ± 4.7	74.0 ± 2.8	0.504
	RVD (mm)		52.2 ± 15.3	56.1 ± 16.0	48.3 ± 14.2	0.222
	LAD (mm)		53.5 ± 9.1	54.8 ± 6.4	52.3 ± 11.4	0.514
	RAD1 (mm)		62.7 ± 8.8	61.8 ± 9.3	63.6 ± 8.7	0.638
	RAD2 (mm)		49.2 ± 6.2	48.0 ± 5.0	50.3 ± 7.3	0.372
	PASP (mmHg)		55.0 ± 18.6	56.0 ± 19.2	54.1 ± 18.7	0.807
	NT-proBNP (pg/mL)		8511 (3528–17,593)	4239 (2487–16,422)	10,710 (8388–17,593)	0.045
		>8200 pg/mL	13 (54.2)	3 (25.0)	10 (83.3)	0.004
	UA (µmol/L)		598 (431–650)	470 (324–632)	609 (563–682)	0.060
Change (∆) and percentage change in parameters over the 2-year pharmacological treatment before HTx						
∆ LVEF (%)			–2.00 (–3.75 to 1.00)	0.50 (–2.00 to 2.00)	–3.00 (–6.50 to –1.25)	0.024
	percentage change		–8.0 (–13.5 to 4.9)	2.4 (–8.5 to 11.1)	–12.0 (–22.1 to –5.7)	0.028
∆ LVD (mm)			5.00 (2.00 to 8.00)	6.00 (2.00 to 8.00)	5.00 (2.00 to 7.50)	0.551
	percentage change		7.6 (2.8 to 11.5)	8.9 (3.0 to 12.5)	7.2 (2.8 to 10.8)	0.378
∆ RVD (mm)			3.50 (1.00 to 8.00)	5.50 (1.25 to 10.75)	2.00 (1.00 to 6.75)	0.347
	percentage change		9.3 (2.4 to 18.0)	12.0 (1.7 to 24.7)	6.2 (2.5 to 16.1)	0.410
∆ LAD (mm)			4.00 (1.25 to 5.00)	4.00 (2.25 to 5.75)	2.50 (–0.75 to 5.00)	0.198
	percentage change		7.5 (2.1 to 10.4)	8.8 (4.3 to 12.0)	4.7 (–1.3 to 9.5)	0.128
∆ RAD1 (mm)			4.00 (–0.75 to 8.00)	2.50 (–0.75 to 9.00)	4.00 (–0.50 to 10.25)	1.000
	percentage change		6.8 (–0.9 to 13.6)	4.2 (–0.9 to 16.5)	7.7 (–3.2 to 13.6)	1.000
∆ RAD2 (mm)			2.50 (–0.75 to 6.00)	2.50 (0.25 to 5.50)	2.00 (–3.25 to 8.25)	0.887
	percentage change		5.2 (–1.7 to 16.9)	5.2 (0.5 to 13.1)	4.3 (6.0 to 19.4)	0.932
∆ PASP (mmHg)			6.00 (–0.50 to 15.75)	9.50 (–5.75 to 22.50)	3.00 (–0.50 to 10.25)	0.266
	percentage change		17.7 (–0.4 to 34.0)	23.7 (–10.1 to 49.6)	6.6 (–0.4 to 19.5)	0.143
∆ NT-proBNP (pg/mL)			–86.5 (–4030.7 to 2341.5)	–86.5 (–1473.0 to 673.7)	–1696.0 (–8830.7 to 7082.5)	0.755
	percentage change		–1.8 (–39.8 to 25.2)	–1.8 (–32.8 to 25.2)	–9.9 (–51.9 to 56.6)	0.514
∆ UA (µmol/L)			–82.5 (–181.7 to –3.2)	–51.0 (–133.5 to 32.5)	–94.5 (–197.2 to –39.0)	0.052
	percentage change		–12.3 (–22.7 to –0.7)	–9.1 (–22.0 to 10.3)	–12.9 (–23.0 to –6.1)	0.198

Adverse events were defined as left ventricular systolic function worsening (LVEF, n = 3), cardiac chambers enlargement (50% increase in RVD/LVD over time, n = 12), or death (n = 2) during follow-up. ACEI, angiotensin converting enzyme inhibitors; ALT, alanine aminotransferase; AST, aspartate aminotransferase; CRP, C-reactive protein; cTNI, cardiac troponin I; Cr, serum creatinine; DCM, dilated cardiomyopathy; HTx, heart transplantation; LAD, end-systolic left atrial anterior–posterior diameter; LDL-C, low-density lipoprotein cholesterol; LVD, end-diastolic left ventricular diameter; LVEF, left ventricular ejection fraction; NT-proBNP, N-terminal pro-brain natriuretic peptide; PASP, pulmonary artery systolic pressure; RAD1, end-systolic right atrial long-axis diameter; RAD2, end-systolic right atrial short-axis diameter; RVD, end-diastolic right ventricular middle diameter; TC, total cholesterol; TG, triglyceride; UA, uric acid.

### 3.2 Serial Changes in Echocardiographic Measures, NT-proBNP, and UA Post-HTx

Table 2 illustrates the sequential changes in echocardiographic measures, NT-proBNP, and UA for the entire HTx cohort. Notably, LVD and RVD at 36 months post-HTx exhibited a significant increase compared to measurements at 3 months post-HTx. The most notable enlargement occurred in RVD (T1: 32.0 mm *vs.* T2: 32.3 mm *vs.* T3: 36.3 mm *vs.* T4: 42.3 mm, *p* = 0.002). LVEF and PASP exhibited a slight reduction over time, while RA dimensions remained unchanged over the observation period. Serum NT-proBNP levels were slightly reduced, while UA levels remained constant.

**Table 2.** **Serial changes in echocardiographic measurements, NT-proBNP, and UA post-HTx in the entire HTx cohort**.

	T0	T1	T2	T3	T4	*p* value
	7 days	3 months	6 months	12 months	36 months	
	post-HTx	post-HTx	post-HTx	post-HTx	post-HTx	
	Mean	Estimated marginal mean (95% CI)	Estimated marginal mean (95% CI)	Estimated marginal mean (95% CI)	Estimated marginal mean (95% CI)	
LVEF (%)	62.7	60.8 (60.2–61.5)	60.9 (59.1–62.7)	59.8 (57.3–62.3)	57.6 (54.1–61.1)‡	0.028
LVD (mm)	43.1	42.9 (41.7–44.0)	43.1 (41.8–44.5)	44.7 (42.8–46.6)	46.7 (43.8–49.6)*	0.051
RVD (mm)	30.9	32.0 (29.8–34.2)	32.3 (29.8–34.8)	36.3 (32.6–39.9)†	42.3 (37.2–47.4)*†‡	0.002
LAD (mm)	38.3	39.4 (36.8–42.0)	38.9 (37.6–40.2)	38.5 (37.0–40.0)	40.9 (38.4–43.4)‡	0.047
RAD1 (mm)	45.4	44.3 (43.1–45.5)	44.4 (43.1–45.6)	43.9 (41.8–46.1)	45.2 (42.8–47.6)	0.161
RAD2 (mm)	35.4	34.0 (32.6–35.4)	34.0 (32.5–35.4)	33.5 (31.7–35.2)	35.2 (33.2–37.3)	0.235
PASP (mmHg)	33.0	32.1 (29.8–34.3)	31.4 (29.4–33.4)	29.7 (27.9–31.6)	30.6 (28.4–32.7)	0.088
Ln NT-proBNP	6.86	5.76 (5.54–6.00)	5.31 (4.86–5.75)	4.79 (4.15–5.44)*	4.86 (4.03–5.70)	0.053
UA (µmol/L)	482	467 (435–499)	460 (421–499)	461 (425–497)	440 (403–476)	0.283

One-way repeated measures analysis of variance (ANOVA) was conducted using the general linear model, with measures at 7 days post-HTx (T0) as covariates appearing in the models. * *p <* 0.05 *vs.* T1, † *p* < 0.05 *vs.* T2, ‡ *p* < 0.05 *vs.* T3. 95% CI, 95% confidence interval; HTx, heart transplantation; LAD, end-systolic left atrial anterior–posterior diameter; LVEF, left ventricular ejection fraction; LVD, end-diastolic left ventricular diameter; Ln NT-proBNP, natural logarithmic transformed N-terminal pro-brain natriuretic peptide; PASP, pulmonary artery systolic pressure; RAD1, end-systolic right atrial long-axis diameter; RAD2, end-systolic right atrial short-axis diameter; RVD, end-diastolic right ventricular middle diameter; UA, uric acid.

**Fig. 2.**
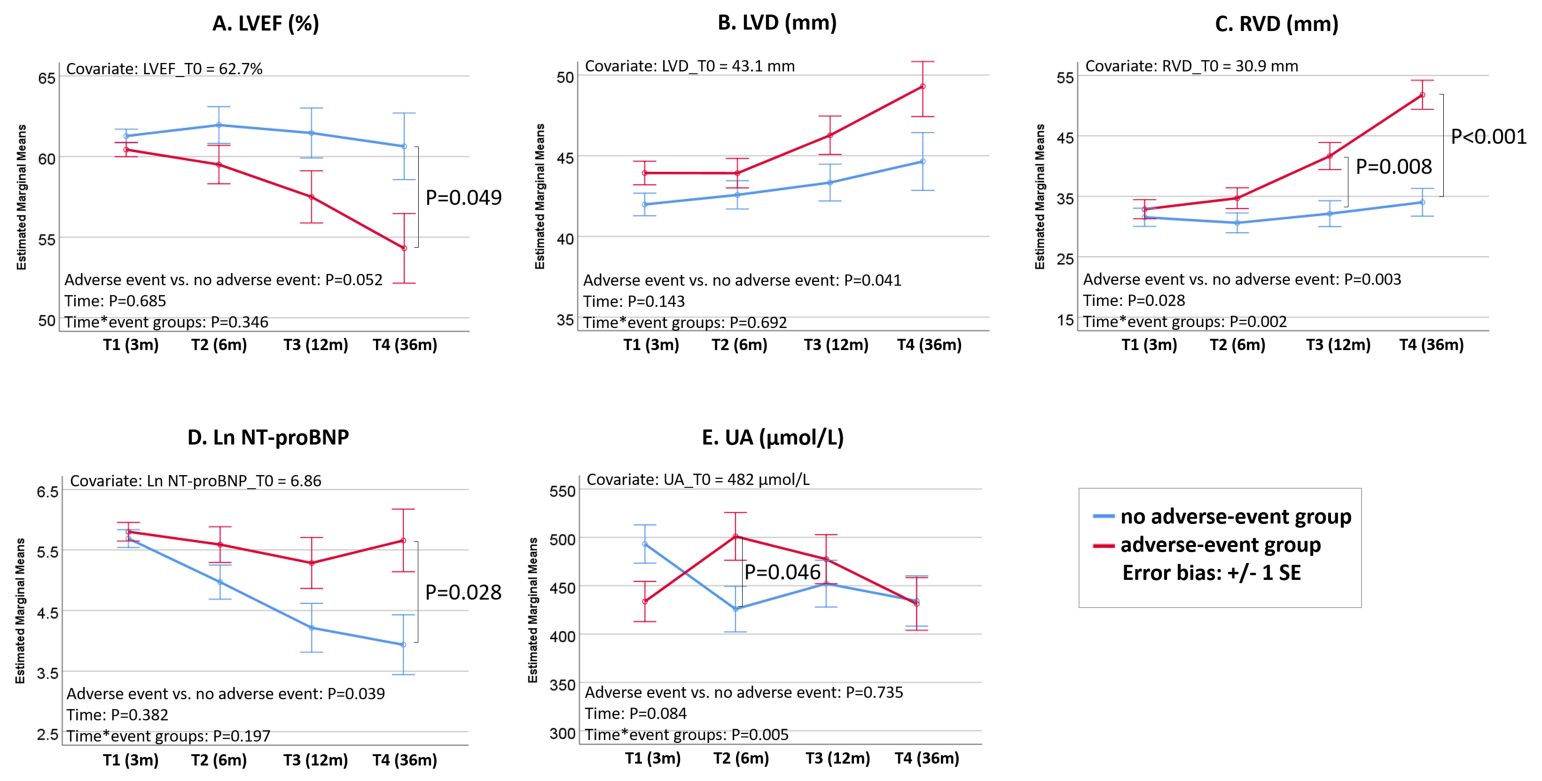
**Bar plots with estimated marginal mean ± 1 standard error (SE), illustrating dynamic change in LVEF (A), LVD (B), RVD (C), Ln NT-proBNP (D), and UA (E) at 3 months (T1), 6 months (T2), 12 months (T3), and 36 months (T4) post-HTx in DCM patients with and without adverse events**. DCM, dilated cardiomyopathy; HTx, heart transplantation; Ln NT-proBNP, natural logarithmic transformed N-terminal pro-brain natriuretic peptide; LVD, end-diastolic left ventricular diameter; LVEF, left ventricular ejection fraction; RVD, end-diastolic right ventricular middle diameter; T0, at 1 week post-HTx; UA, uric acid.

As depicted in Fig. 2, HTx patients in the adverse-event group exhibited a notable decrease in LVEF, a significant increase in RVD, and a sustained NT-proBNP level. Conversely, HTx patients in the non-adverse-event group demonstrated stable LVEF, LVD, and RVD, coupled with a significant reduction in NT-proBNP levels.

**Fig. 3.**
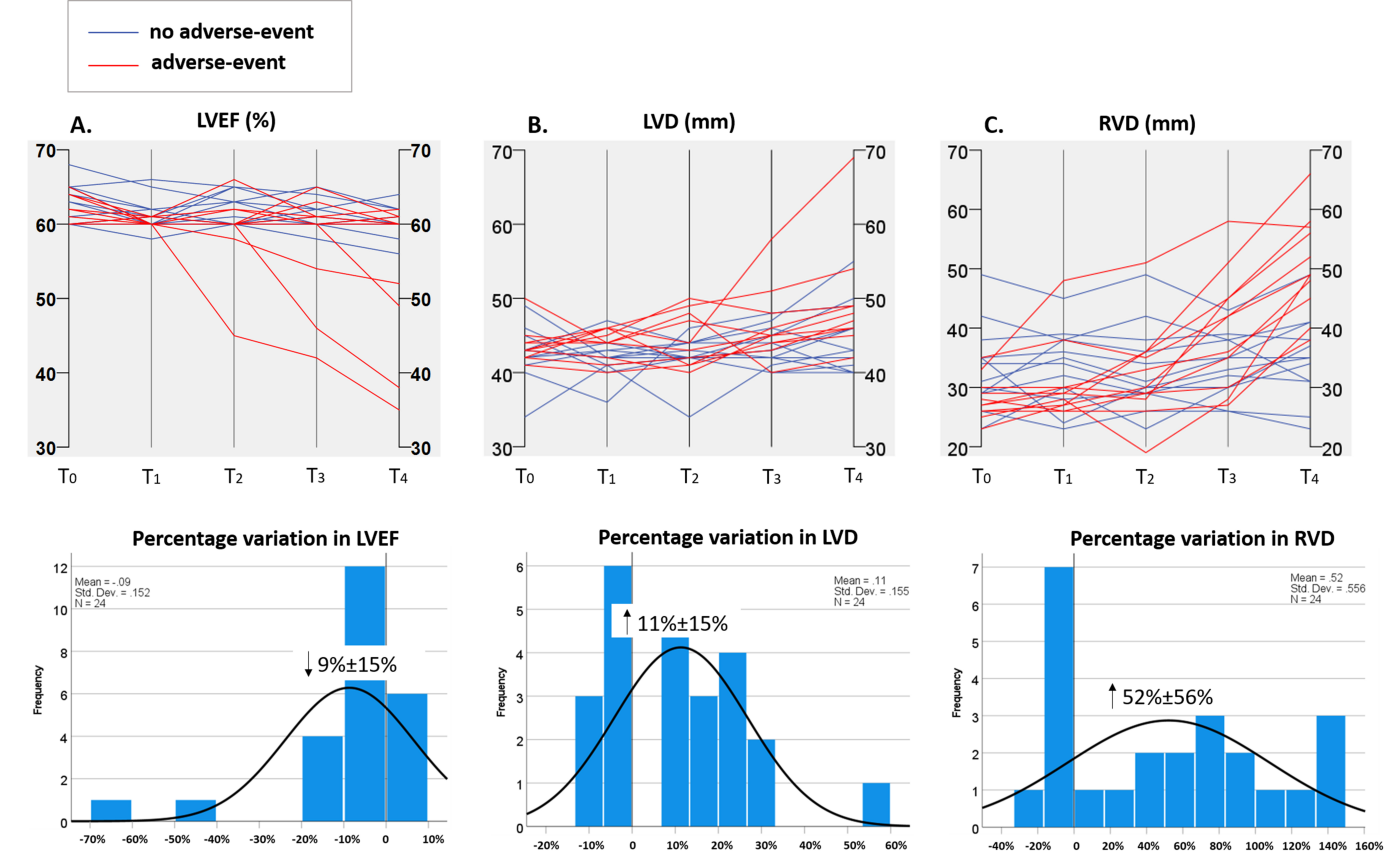
**Dynamic changes and percentage variations (%, mean ± standard deviation) in LVEF (A), LVD (B), and RVD (C) from 1 week (T0) to 36 months (T4) post-HTx in DCM patients**. LVD, end-diastolic left ventricular diameter; LVEF, left ventricular ejection fraction; RVD, end-diastolic right ventricular middle diameter; T1, 3 months post-HTx; T2, 6 months post-HTx; T3, 12 months post-HTx; ↑, represents an increase; ↓, represents a reduction; HTx, heart transplantation; DCM, dilated cardiomyopathy.

**Fig. 4.**
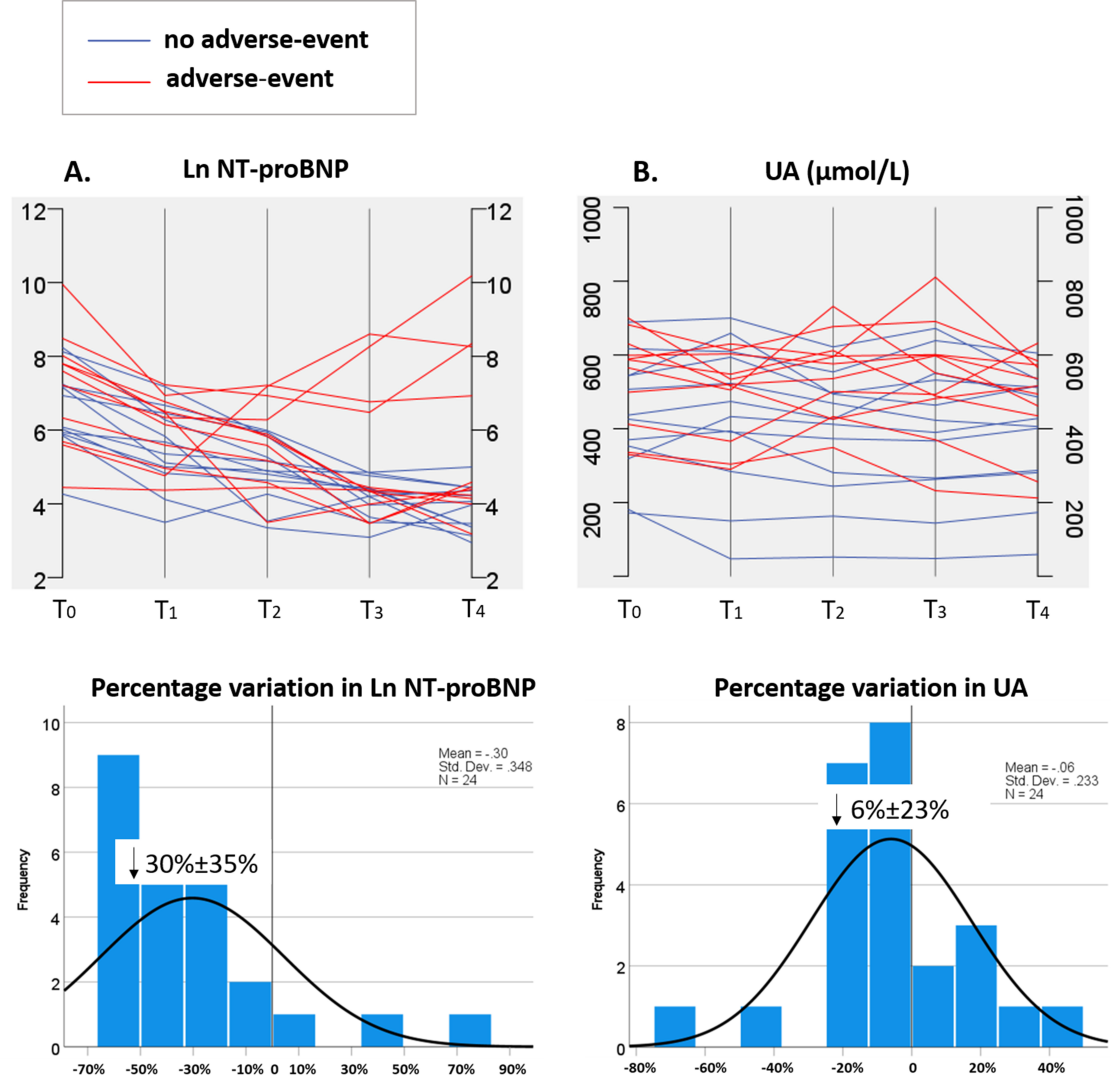
**Dynamic changes and percentage variations (%, mean ± standard deviation) in Ln NT-proBNP (A) and UA (B) from 1 week (T0) to 36 months (T4) post-HTx in DCM patients**. Ln NT-proBNP, natural logarithmic transformed N-terminal pro-brain natriuretic peptide; T1, 3 months post-HTx; T2, 6 months post-HTx; T3, 12 months post-HTx; UA, uric acid; ↓, represents a reduction; HTx, heart transplantation; DCM, dilated cardiomyopathy.

Figs. 3,4 depict the chronological alterations in echocardiographic measures, NT-proBNP, and UA for individual patients and the percentage variations in these parameters. When observing the overall trends, LVEF showed a gradual decrease (–9%), and NT-proBNP exhibited a consistent decline (–30%), while RVD increased (52%) and LVD showed a gradual rise (11%) over time post-HTx.

### 3.3 Independent Prognostic Factors for Adverse Events Post-HTx

Modified Poisson Log-linear models were employed to identify the independent prognostic significance of baseline NT-proBNP, UA level, and LVEF deterioration during pharmacological treatment prior to HTx for adverse events post-HTx (Table 3). After adjusting for age, sex, and baseline LVEF, HTx patients with baseline NT-proBNP levels >7390 pg/mL were associated with an approximately 7-fold increased risk in adverse events compared to those with baseline NT-proBNP levels ≤7390 pg/mL (event rates: 78.6% *vs.* 10.0%, *p* = 0.003; RR = 7.412, 95% CI = 1.034–53.132, *p* = 0.046). Similarly, a baseline UA level >542 µmol/L was associated with an almost 9-fold increased risk of adverse events compared to a baseline UA level of ≤542 µmol/L (event rates: 73.3% *vs.* 11.1%, *p* = 0.009; RR = 8.838, 95% CI = 1.541–50.694, *p* = 0.014). Additionally, LVEF deterioration during the 2-year pharmacological treatment prior to HTx (reduction ≥3%) was linked to a 3-fold increased risk adverse events compared to a stable LVEF (event rates: 33.3% *vs.* 0.0%; RR = 3.252, 95% CI = 1.240–8.532, *p* = 0.017).

**Table 3.** **Adjusted prognostic performance of baseline NT-proBNP, UA, and LVEF deterioration before heart transplantation for predicting combined adverse events**.

	Event rates (%)	*p* value	Age, sex, and baseline LVEF adjusted RR (95% CI)	*p* value
Baseline NT-proBNP >7390 *vs.* ≤7390 pg/mL	78.6 *vs.* 10.0	0.003	7.412 (1.034–53.132)	0.046
Baseline UA >542 *vs.* ≤542 µmol/L	73.3 *vs.* 11.1	0.009	8.838 (1.541–50.694)	0.014
LVEF reduction ≥3% *vs.* <3% within 2 years before HTx	33.3 *vs.* 0.0	0.118	3.252 (1.240–8.532)	0.017

Modified Poisson Log-linear models were employed to identify independent risk factors linked to adverse outcomes. 95% CI, 95% confidence interval; HTx, heart transplantation; LVEF, left ventricular ejection fraction; NT-proBNP, N-terminal pro-brain natriuretic peptide; RR, relative risk; UA, uric acid.

## 4. Discussion

The present study demonstrates that elevated baseline levels of NT-proBNP (>7390 pg/mL), UA (>542 µmol/L), and LVEF deterioration during the 2-year pharmacological treatment prior to HTx (reduction ≥3%) are associated with an increased risk of adverse events post-HTx in young adult DCM patients. Notably, these factors also function as independent determinants of adverse events post-HTx in this patient cohort.

To the best of our knowledge, this is the first clinical report delineating independent risk factors preceding HTx for adverse ventricular remodeling in young adult patients with DCM post-HTx. Although the precise pathophysiological mechanisms remain unclear, our data suggest a potential association between higher baseline levels of NT-proBNP and UA and adverse ventricular remodeling post-HTx in young adults with DCM.

### 4.1 Association between Change in LVEF Prior HTx and Outcome Post-HTx

Numerous studies on DCM have consistently revealed a positive correlation between the decrease in LVEF and adverse outcomes, such as all-cause mortality, HTx, sudden cardiac death, and major ventricular arrhythmias [14, 15]. Existing data indicate that a dynamic decline in LVEF among DCM patients, even with optimal medication, is associated with an increased risk of cardiac events, encompassing death, HTx, or major ventricular arrhythmias [16, 17, 18]. Gentile *et al*. [16] observed a significantly higher risk of long-term major events in patients with mid-range ejection fraction (HFmrEF, LVEF 40–49%) transitioning to reduced ejection fraction (HFrEF, LVEF <40%) compared to baseline HFrEF patients, over a median follow-up of 120 months. Manca *et al*. [18] demonstrated a sharp increase in the risk of all-cause death, HTx, or left ventricular assist device for each point of LVEF decline up to 8%, compared to patients with stable LVEF.

Despite the wealth of data on the impact of LVEF changes in DCM patients, there is limited information on how pre-HTx LVEF changes influence post-HTx outcomes in this population. Our study fills this gap by revealing that LVEF reduction over a 2-year pharmacological treatment period before HTx is associated with a higher likelihood of adverse outcomes in young adult DCM patients post-HTx. The observed association between LVEF deterioration before HTx and worse outcomes in HTx patients may be indicative of an advanced stage of DCM with heightened myocardial damage. The subsequent compromised cardiac function could pose challenges in adapting to the stresses of the transplantation procedure. A recent study utilizing the Spanish National Heart Transplant Registry revealed that recipients categorized as the Interagency Registry for Mechanically Assisted Circulatory Support (INTERMACS) profile 1 (critical cardiogenic shock) and profile 2 (progressive clinical decline despite inotrope treatment) faced elevated risks of primary graft failure, dialysis need, and in-hospital mortality [19]. The dynamic decline in LVEF may also indicate inherent myocardial vulnerabilities, increasing susceptibility to ischemic insults, immune reactions, or other post-transplant stressors. Although the underlying mechanism remains elusive, our findings emphasize the need for vigilant monitoring, especially in the postoperative period, for patients exhibiting dynamic LVEF decline before HTx.

### 4.2 Impact of Baseline NT-proBNP on Post-HTx Outcomes

Ventricular remodeling, a fundamental pathological process in heart failure following acute myocardial infarction (AMI) or DCM, substantially increases the risk of cardiac death [17, 20]. Prolonged remodeling negatively influences cardiac function, leading to notable morbidity and mortality. Serum NT-proBNP levels have been recognized as a sensitive marker for predicting ventricular remodeling in AMI and DCM patients [21, 22]. Several studies have demonstrated the independent predictive value of serum NT-proBNP in ventricular remodeling for heart failure (HF) following AMI [23] and in children with HF secondary to DCM [22]. NT-proBNP levels exceeding 1000 pg/mL can be used to identify symptomatic children. Additionally, Temporelli *et al*. [24] affirmed that preoperative NT-proBNP assessments (coronary artery bypass grafting) aid in evaluating postoperative LVEF and ventricular remodeling.

The clinical utility of NT-proBNP in HTx remains inadequately documented and has yielded controversial conclusions. Previous investigations into the relationship between NT-proBNP concentrations and survival post-HTx have presented mixed findings. Combining NT-proBNP and C-reactive protein as markers of acute rejection can significantly enhance their predictive value for developing cardiac allograft vasculopathy (CAV) and all-cause mortality during the first year post-HTx [25]. Moreover, research by Avello *et al*. [26] suggests that serial measurements of NT-proBNP are crucial for the proper follow-up of HTx patients. In fact, all patients exhibiting rejection showed a significant increase in NT-proBNP concentration compared to their previous values. The authors propose a serum NT-proBNP concentration of 1000 ng/L as a potential cutoff value for classifying patients at risk of death during the year following the analysis. However, a recent systematic review and meta-analysis by Zhu *et al*. [9] cast doubt on the reliability of serum BNP and NT-proBNP, suggesting insufficient sensitivity and specificity for predicting adverse outcomes following HTx.

Our study revealed that patients experiencing adverse events more frequently exhibited elevated baseline NT-proBNP levels compared to those in the non-adverse-event group. Baseline NT-proBNP levels >7390 pg/mL remain an independent risk factor for combined adverse events in young adult patients with DCM undergoing HTx. The activation of the natriuretic peptide B (*BNP*) *gene* in response to myocardial stress, primarily induced by stretching, leads to the production of both BNP and NT-proBNP peptides [27]. Elevated baseline NT-proBNP levels in DCM patients undergoing HTx may signify persistent myocardial stress and dysfunction, reflecting a more severe state of cardiac impairment. This prolonged stress on the myocardium could contribute to ongoing pathological processes and hinder the adaptability of the transplanted heart. Additionally, higher baseline NT-proBNP levels may indicate pre-existing irreversible cardiac damage, potentially making the heart more susceptible to post-HTx complications.

### 4.3 Impact of Baseline UA on Post-HTx Outcomes

Previous research has consistently demonstrated a robust association between elevated UA concentrations and ventricular remodeling [28, 29]. Liu *et al*. [28] found that high levels of serum UA were associated with an increased risk of LV hypertrophy, end-diastolic LV internal diameter enlargement, and LVEF reduction in patients with coronary heart disease. Elevated UA levels are known to stimulate excessive production of oxygen free radicals within cells, leading to endothelial injury. Moreover, high serum UA levels can activate the renin–angiotensin system, contributing to vascular endothelial dysfunction [30, 31]. These changes may persist and contribute to the adverse outcomes observed in our patients. Supporting this hypothesis, Chen *et al*. [29] demonstrated that elevated serum UA levels were associated with unfavorable ventricular remodeling, and increased myocardial oxidative stress might promote the development of adverse ventricular remodeling, potentially through a superoxide and endothelin-1-dependent pathway.

Previous study has highlighted the prognostic significance of UA in patients post-HTx. Kittleson *et al*. [32] reported that elevated baseline UA levels were linked to an increased risk of CAV among heart transplant recipients during a median follow-up of 5 years post-HTx. Similarly, Asleh *et al*. [33] suggested that baseline UA levels independently predicted the incidence of CAV post-HTx. Consistent with these findings, our study observed a correlation between baseline UA levels and adverse outcomes in young DCM patients post-HTx.

Notably, LVEF, baseline serum levels of NT-proBNP and UA are integral components in Heart Failure Prognosis Scores used in the HTx listing criteria [34]. Our study reveals that these key indicators, commonly employed for HTx eligibility assessments, may also hold value in evaluating the risk of adverse events post-HTx among young DCM patients.

### 4.4 Clinical Implication

Our study underscores the importance of intensified post-HTx monitoring for young DCM patients who present with elevated baseline levels of NT-proBNP and UA, along with a reduction in LVEF within the 2 years prior to HTx. These specific baseline features are crucial indicators for heightened vigilance during the post-HTx period. Developing and implementing targeted monitoring strategies tailored to these identified risk factors can significantly enhance the overall post-HTx outcomes for this patient cohort.

### 4.5 Limitations

The current study has several limitations. It is a retrospective, non-randomized, and single-center study, potentially affecting the generalizability of the findings. Additionally, the patient cohort is relatively small, thereby limiting the statistical power of the study. Larger-scale studies are necessary to validate and strengthen our observed associations. Lastly, the precise pathophysiological mechanisms underlying the identified associations, particularly regarding baseline NT-proBNP, UA, and LVEF deterioration before HTx, remain largely unclear. Future investigations are crucial for a more in-depth understanding of these mechanisms and their impact on outcomes post-HTx.

## 5. Conclusions

In this study, conducted with a limited number of DCM patients, we found that elevated baseline NT-proBNP (>7390 pg/mL), elevated UA (>542 µmol/L), and LVEF reduction (≥3%) during the 2-year pharmacological treatment period before HTx are significantly linked to an increased risk of adverse events in young adult DCM patients post-HTx. Confirmation of these findings and the exploration of whether more intensive monitoring strategies can enhance outcomes for these high-risk patients post-HTx necessitate further investigation in larger patient cohorts.

## Data Availability

Data are available on reasonable request (contact the corresponding author Dr. Junhua Ge).

## Abbreviations

ALT, alanine aminotransferase; AST, aspartate aminotransferase; AMI, acute myocardial infarction; BNP, natriuretic peptide B; CAV, cardiac allograft vasculopathy; CRP, C-reactive protein; cTNI, cardiac troponin I; Cr, serum creatinine; DCM, dilated cardiomyopathy; HTx, heart transplantation; ISHLT, International Society of Heart and Lung Transplantation; LAD, end-systolic left atrial anterior–posterior diameter; LDL-C, low-density lipoprotein cholesterol; LVD, end-diastolic left ventricular diameter; LVEF, left ventricular ejection fraction; NT-proBNP, N-terminal pro-brain natriuretic peptide; PASP, pulmonary artery systolic pressure; RAD1, end-systolic right atrial long-axis diameter; RAD2, end-systolic right atrial short-axis diameter; RVD, end-diastolic right ventricular middle diameter; TC, total cholesterol; TG, triglyceride; UA, uric acid.
